# Disparities in Medicare Annual Wellness Visits After a Systemwide Quality Improvement Initiative: A Serial Cross-sectional Analysis

**DOI:** 10.1007/s11606-025-09773-3

**Published:** 2025-07-25

**Authors:** David T. Liss, Ji Young Lee, Tiffany Brown, Gayle Kricke, Tahniat Nadeem, Jeffrey A. Linder

**Affiliations:** 1 Division of General Internal Medicine, Northwestern University Feinberg School of Medicine, Chicago, IL USA; 2 AllianceChicago, Chicago, IL USA; 3 Northwestern Medicine, Chicago, IL USA

**Keywords:** Preventive care, Preventive visits, Medicare, Disparities, Health equity, Program evaluation

## Abstract

**Background:**

Medicare Annual Wellness Visits (AWVs) offer potential benefits for older adults. Little is known about recent racial/ethnic disparities in populations with high AWV completion.

**Objective:**

To calculate racial/ethnic disparities before and after a health system initiative to increase AWV completion, and investigate factors associated with post-initiative AWV completion.

**Design:**

Repeated cross-sectional evaluation over 3 years, ending August 2023.

**Patients:**

Medicare beneficiaries age ≥ 65 in a large Midwestern U.S. health system. Race/ethnicity was categorized as White non-Hispanic, Black/African American non-Hispanic, Latino/Hispanic, Asian non-Hispanic, and Other non-Hispanic.

**Main Measures:**

Annual AWV completion during the pre-initiative year (year 0) and two subsequent initiative years. Regression models tested for unadjusted and covariate-adjusted disparities in AWV completion.

**Key Results:**

The health system’s overall rate of AWV completion increased from 47.5% in year 0 to 68.9% in year 2. All racial/ethnic groups experienced similar proportional increases in AWV completion, including 21.5% among White, 21.6% among Black, and 22.5% among Latino/Hispanic patients. In unadjusted regression for year 2 (*N* = 92,634) results, the probability of AWV completion was 15% lower among both Black (relative risk, [RR], 0.85; 95% confidence interval [CI], 0.82–0.89) and Latino/Hispanic (RR, 0.85; 95% CI, 0.82–0.87) patients, compared to Whites. However, the adjusted probability of AWV completion was only 4% lower in Black (RR, 0.96; 95% CI, 0.93–1.00) and 5% lower in Latino/Hispanic (RR, 0.95; 95% CI, 0.91–0.99) patients than in Whites. AWV completion was positively associated with age ≥ 70, using the patient portal for appointment scheduling, and prior year AWV completion, and negatively associated with Medicaid coverage, positive screening for any social need, and dementia.

**Conclusions:**

A 2-year health system initiative led to substantially increased AWV completion, but pre-existing disparities persisted. To reduce disparities, future efforts should explicitly focus on enhancing equity and addressing barriers in subgroups with particularly low AWV completion.

**Supplementary Information:**

The online version contains supplementary material available at 10.1007/s11606-025-09773-3.

## INTRODUCTION

Checkup visits—also known as general health checks, preventive visits, and wellness visits, among other terms—can increase preventive service completion^1–4^ and improve a variety of patient-reported outcomes^1,2,5–7^ such as self-rated health,^1^ which is predictive of mortality.^8,9^ Additionally, though checkups have not been shown to consistently decrease mortality in all adults,^10^ the two randomized trials where checkups reduced mortality were conducted in older adults.^11,12^ Given the substantial potential benefits of these visits, there is clear justification for efforts to maximize checkup uptake in older populations such as patients insured through Medicare.

Among older Americans, the Medicare Annual Wellness Visit (AWV) is the most common type of checkup; since 2011, Medicare beneficiaries have been eligible for an AWV each year at zero copay. However, despite the potential wide-ranging benefits of these visits, their uptake has proceeded unevenly and unequally. In 2014, only 16% of Medicare beneficiaries completed AWVs,^13^ and 23% did so in 2016.^14^ AWV completion was also more likely among non-Hispanic White and relatively affluent patients, with Black/African American Medicare beneficiaries consistently completing AWVs less often than Whites in both unadjusted^13–15^ and adjusted^13,14^ analyses. Similarly, Latino/Hispanic beneficiaries had lower unadjusted rates of AWV completion than non-Hispanic Whites,^16^ but differences did not persist after covariate adjustment.^14,16^ Additionally, beneficiaries with lower education or income (at both the patient^16^ and zip code^15^ level) completed AWVs at lower rates.

As AWV adoption (by physicians) and AWV completion (by patients) has substantially increased over time,^17^ research is now needed to enhance our understanding of current disparities. During the 5-year period after AWVs were established (2011–2015), many patients had limited if any access to AWVs, as a majority of primary care practices did not deliver any AWVs.^18^ However, the number of physicians adopting AWVs more than doubled between 2012 and 2019,^17^ and by 2020 nearly half of Medicare beneficiaries were completing AWVs.^19^ In the current era of relatively high AWV access and completion, new studies should also account for a variety of factors that may contribute to disparities, including comorbidity burden,^17^ health-related social needs,^20,21^ and technology use.

This study investigated factors associated with AWV completion in a population that experienced substantial increases in AWV completion. In the health system under study, an initiative to increase AWV completion began in late 2021. While large aggregate increases in AWV completion were observed during both years of the initiative, there has not yet been an in-depth investigation of detailed patient-level data, particularly with a lens toward racial and ethnic disparities.

This study had two aims. First, we sought to describe racial/ethnic disparities in AWV completion, both before and after the health system initiative under study. Second, we sought to investigate factors associated with post-initiative AWV completion. We hypothesized that racial/ethnic disparities would be observed both before and after the initiative, but that several factors—such as comorbidity burden, social risks, and primary care utilization for non-AWV visits—would also be associated with AWV completion after covariate adjustment.

## METHODS

### Study Design and Setting

This study was a serial cross-sectional evaluation of three annual waves of data from Northwestern Medicine (NM), a large academic health system in Illinois. Study protocols were approved by Northwestern University’s institutional review board, with a waiver of informed consent (study #219825).

NM’s system-level initiative to promote AWV completion began in September 2021. Several workflow changes targeted older adults who were due or overdue for a yearly Medicare AWV, such as electronic reminders sent to registered patient portal users, and opportunistic AWV scheduling at the point of care (e.g., during checkout at the end of in-person visits, clinic staff asked eligible patients if they wanted to schedule a future AWV). Patients were defined as achieving AWV completion if they completed an Initial Preventive Physical Exam (i.e., the “Welcome to Medicare” visit), an initial AWV, or subsequent AWV. The initiative is described in greater detail in Appendix Table 3.

Data was collected for each of three 1-year periods between September 2020 and August 2023. The pre-initiative baseline year (year 0) began on September 1, 2020, and ended August 31, 2021. Data from subsequent 12-month periods represented the two respective years of the initiative (year 1; year 2).

### Patients

Patients were included in each year they met inclusion criteria. Patients were required to be Medicare or Medicare Advantage beneficiaries, and age 65 years or greater on the first day of year 2. Annual inclusion was determined by whether each patient made one or more in-person primary care visits during the prior 18 months (e.g., for the year 2 period between September 2022 and August 2023, patients were required to have at least one primary care encounter between March 2021 and August 2022). Patients were excluded from any years they were age 64 or lower.

### Variables

Beginning in February 2024, study data were collected from the Northwestern Medicine Enterprise Data Warehouse (NMEDW), an institutional repository containing data on insurance, clinical encounters and diagnoses, demographic characteristics, screenings, and MyChart patient portal (Epic Systems Corporation; Verona, WI) data. Each patient’s primary care physician was identified as the physician seen most frequently during the 18 months preceding each year (in the case of a tie, the physician seen most recently was selected).

The primary outcome was a binary measure (0/1) of Medicare AWV completion during each year. Each patient was defined as completing a Medicare AWV if, during the year, any of three Current Procedural Terminology (CPT) codes for Medicare preventive visits were documented (G0402, Initial Preventive Physical Examination; G0438, initial Medicare AWV; G0439, subsequent Medicare AWV).

Patients’ self-reported race and ethnicity (as reported to registration) were combined into a single race/ethnicity variable, in accordance with recommended best practices^22^ aligning with self-identification practices of many Latino individuals.^23^ The five defined race/ethnicity groups were White non-Hispanic, Black/African American non-Hispanic, Latino/Hispanic (any race), Asian non-Hispanic, and Other non-Hispanic (henceforth White, Black, Latino/Hispanic, Asian, and Other). Prior to defining combined race/ethnicity categories, we examined the reported race of included patients with Latino/Hispanic ethnicity; less than 2% reported Asian race, and less than 1% reported Black/African American race.

We extracted covariate measures for patient characteristics including sex at birth and primary language (English vs. non-English). Patients’ age on the first day of each year was stratified into four categories (65–69; 70–74; 75–79; ≥ 80). To measure overall comorbidity burden, we calculated each patient’s Charlson comorbidity index score at the beginning of each year,^24^ and whether the patient had a pre-existing diagnosis of dementia on their problem list. A three-category variable captured prior year AWV completion; patients meeting inclusion criteria during the preceding year were categorized based on AWV completion during the prior year (no; yes), while those not meeting inclusion criteria during the preceding year were categorized as “excluded.” Insurance coverage was defined by two binary variables: type of Medicare coverage (traditional Medicare vs. Medicare Advantage), and Medicaid coverage in addition to Medicare.

We also captured factors potentially associated with concurrent AWV completion. Concurrent primary care utilization was defined using the count of primary care visits (excluding AWVs) each year. Owing to very high patient portal use (89.1%) in the study population, a measure of whether each patient used the portal to schedule a clinical appointment each year was collected as a proxy of technology usage. During the study period, the health system began population-based screening for health-related social needs such as food insecurity, housing instability, transportation, and ability to afford medications. Social needs screenings were conducted via phone, MyChart, or in person for patients with primary care visits (including AWVs) or hospital-based encounters (e.g., inpatient admissions). Patients were categorized as having screened negative (i.e., zero social needs), screened positive for any needs, or not screened. Additionally, the health system is divided into five administrative regions; the NM region where each patient received primary care accounted for administrative and geographic variation.

### Statistical Analysis

In patient-level analyses, we calculated frequencies of patient characteristics variables for each year. Year 2 patient characteristics were additionally stratified by race/ethnicity. Unadjusted racial/ethnic differences in categorical patient characteristics were evaluated via chi-square tests. Proportions of patients achieving the primary outcome—annual AWV completion—were calculated overall and by race/ethnicity. Proportional differences in AWV completion were calculated by evaluating differences in annual proportions of patients completing AWVs.

Relative differences in AWV completion were evaluated via two regression models separately testing for unadjusted and adjusted racial/ethnic disparities. Due to the common binary outcome, we estimated Poisson regression models with robust variance estimates, which provide inference on relative risks.^25^ Generalized estimating equations (GEEs) accounted for clustering of patient-level outcomes within primary care physicians.^26^ In our unadjusted regression model, race/ethnicity was the lone independent variable (referent, White). Separately, a multivariable model adjusted for all patient characteristics.

In the first of two sensitivity analyses, we estimated the same multivariable model described above, but did not adjust for year 1 AWV completion. In the second sensitivity analysis, the primary outcome definition was expanded beyond three CPT codes for Medicare preventive visits (G0402, G0438, G0439) to also include two codes for non-Medicare preventive visits in older adults (99387, 99397).

Descriptive results and analysis of differences in patient characteristics were conducted using SAS, version 9.4 (SAS Institute; Cary, NC). Regression analysis was conducted using Stata, version 18.0 (StataCorp; College Station, TX).

## RESULTS

**Figure 1:**
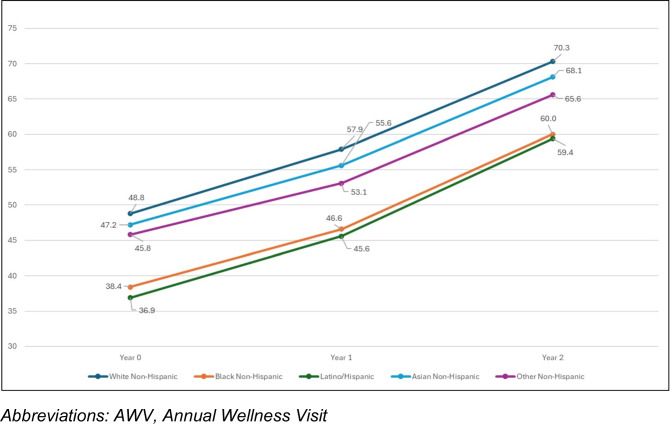
Annual AWV completion, by race/ethnicity

AWV completion increased substantially during the 2-year initiative (Fig. 1). Among all included patients, the rate of AWV completion was 47.5% in year 0, 56.4% in year 1, and 68.9% in year 2 (21.4% total proportional increase). All racial/ethnic groups experienced similar proportional increases in AWV completion during the 2-year initiative, including a 21.5% proportional increase among White patients (48.8% in year 0, 70.3% in year 2), 21.6% among Black patients (38.4% in year 0, 60.0% in year 2), and 22.5% among Latino/Hispanic patients (36.9% in year 0, 59.4% in year 2). As a result, racial/ethnic disparities in AWV proportions that were present during the baseline year were largely unchanged. For example, the proportional difference in AWV completion between White and Black patients was 10.4% in year 0, and 10.3% in year 2; between White and Latino/Hispanic patients, it was 11.9% in year 0 and 10.9% in year 2. Detailed results on annual AWV completion, across patient characteristics, are presented in Appendix Table 4.

During year 2, over four-fifths of included patients (*N* = 92,634) were White race/ethnicity (82.4%), 6.8% were Black, 3.7% were Latino/Hispanic, 2.8% were Asian, and 4.2% were Other race/ethnicity (Table 1, left panel). Sixty percent of included patients were female, and 3.2% spoke a primary language besides English. About one-third of patients had a Medicare Advantage plan, while only 3.9% had dual Medicaid coverage. Similar distributions for these patient characteristics were observed during year 0 and year 1 (Appendix Table 5). In addition, social needs screening—which ramped up substantially during year 2—was completed for 52.8% of included patients; among those who completed screening, most screened negative.

**Table 1:** Patient Characteristics in Year 2, by Race/Ethnicity

Characteristic	Total, no. (column %)*	Race/ethnicity, no. (column %)*				
		**White non-Hispanic**	**Black non-Hispanic**	**Latino/Hispanic**	**Asian non-Hispanic**	**Other non-Hispanic**
N (row %)*^†^	*92,634*	*76,369 (82.4)*	*6321 (6.8)*	*3415 (3.7)*	*2614 (2.8)*	*3915 (4.2)*
Age						
65–69	24,021 (25.9)	19,422 (25.4)	1824 (28.9)	1083 (31.7)	583 (22.3)	1109 (28.3)
70–74	26,397 (28.5)	21,719 (28.4)	1852 (29.3)	930 (27.2)	758 (29.0)	1138 (29.1)
75–79	20,186 (21.8)	16,798 (22.0)	1257 (19.9)	661 (19.4)	603 (23.1)	867 (22.2)
≥ 80	22,030 (23.8)	18,430 (24.1)	1388 (22.0)	741 (21.7)	670 (25.6)	801 (20.5)
Sex						
Female	55,535 (60.0)	45,041 (59.0)	4619 (73.1)	2060 (60.3)	1603 (61.3)	2212 (56.5)
Male	37,099 (40.0)	31,328 (41.0)	1702 (26.9)	1355 (39.7)	1011 (38.7)	1703 (43.5)
Primary language						
English	89,718 (96.9)	75,547 (98.9)	6310 (99.8)	2090 (61.2)	2110 (80.7)	3661 (93.5)
Non-English	2916 (3.2)	822 (1.1)	11 (0.2)	1325 (38.8)	504 (19.3)	254 (6.5)
Charlson comorbidity score						
0–1	17,073 (18.4)	14,228 (18.6)	1051 (16.6)	513 (15.0)	373 (14.3)	908 (23.2)
2	30,882 (33.3)	25,416 (33.3)	1796 (28.4)	1216 (35.6)	1033 (39.5)	1421 (36.3)
3	18,151 (19.6)	14,896 (19.5)	1273 (20.1)	730 (21.4)	530 (20.3)	722 (18.4)
≥ 4	26,528 (28.6)	21,829 (28.6)	2201 (34.8)	956 (28.0)	678 (25.9)	864 (22.1)
Dementia						
No	89,723 (96.9)	74,108 (97.0)	6055 (95.8)	3253 (95.3)	2512 (96.1)	3795 (96.9)
Yes	2911 (3.1)	2261 (3.0)	266 (4.2)	162 (4.7)	102 (3.9)	120 (3.1)
Prior year AWV completion						
No	31,365 (33.9)	25,083 (32.8)	2731 (43.2)	1402 (41.1)	834 (31.9)	1315 (33.6)
Yes	42,923 (46.3)	36,560 (47.9)	2473 (39.1)	1224 (35.8)	1094 (41.9)	1572 (40.1)
Excluded	18,346 (19.8)	14,726 (19.3)	1117 (17.7)	789 (23.1)	686 (26.2)	1028 (26.3)
Primary insurance						
Traditional Medicare	61,881 (66.8)	53,065 (69.5)	2999 (47.4)	1718 (50.3)	1613 (61.7)	2486 (63.5)
Medicare Advantage	30,753 (33.2)	23,304 (30.5)	3322 (52.6)	1697 (49.7)	1001 (38.3)	1429 (36.5)
Medicaid coverage						
No	88,998 (96.1)	74,699 (97.8)	5407 (85.5)	2866 (83.9)	2325 (88.9)	3701 (94.5)
Yes	3636 (3.9)	1670 (2.2)	914 (14.5)	549 (16.1)	289 (11.1)	214 (5.5)
Non-AWV primary care visits						
0	27,540 (29.7)	23,098 (30.3)	1575 (24.9)	750 (22.0)	774 (29.6)	1343 (34.3)
1	25,828 (27.9)	21,488 (28.1)	1585 (25.1)	900 (26.4)	738 (28.2)	1117 (28.5)
2	16,964 (18.3)	13,973 (18.3)	1231 (19.5)	683 (20.0)	433 (16.6)	644 (16.5)
≥ 3	22,302 (24.1)	17,810 (23.3)	1930 (30.5)	1082 (31.7)	669 (25.6)	811 (20.7)
Patient portal used for appointment scheduling						
No	59,378 (64.1)	48,743 (63.8)	4180 (66.1)	2327 (68.1)	1620 (62.0)	2508 (64.1)
Yes	33,256 (35.9)	27,626 (36.2)	2141 (33.9)	1088 (31.9)	994 (38.0)	1407 (35.9)
Health-related social needs						
0 needs	45,911 (49.5)	39,944 (52.3)	2081 (32.9)	1264 (37.0)	1025 (39.2)	1597 (40.8)
≥ 1 need	3036 (3.3)	2271 (3.0)	396 (6.3)	183 (5.4)	78 (3.0)	108 (2.8)
Not screened	43,687 (47.2)	34,154 (44.7)	3844 (60.8)	1968 (57.6)	1511 (57.8)	2210 (56.5)

*AWV* annual wellness visit

^*^Percents may not sum to exactly 100% due to rounding

^†^Row percents presented in the row listing proportions in each racial/ethnic group. All other percents in the table represent column percents within individual patient characteristics

Patient characteristics varied across racial/ethnic groups (Table 1, right panel). Though all chi-square tests were statistically significant at *P* < 0.001, some noteworthy findings included a high proportion of females among Black patients (73.1%, versus ≤ 61.3% in other groups), and high proportions of non-English speakers among Latino/Hispanic (38.8%) and Asian (19.3%) patients (≤ 6.5% in other groups). White patients were most likely to have completed an AWV during the preceding year (47.9%, versus ≤ 41.9% in other groups). Medicare Advantage coverage was most common among Black (52.6%) and Latino/Hispanic (49.7%) patients (≤ 38.3% in other groups). Medicaid coverage was substantially less common among White patients (2.2%) than other racial/ethnic groups (≥ 5.5%). Screening positive for any social needs was more common among Black (6.3%) and Latino/Hispanic (5.4%) patients than other groups (≤ 3.0%).

In the unadjusted regression model, three of four non-White racial/ethnic groups were significantly less likely than Whites to complete AWVs. Compared to White patients, the probability of AWV completion was 15% lower among both Black patients (relative risk [RR], 0.85; 95% confidence interval [CI], 0.82–0.89) and Latino/Hispanic patients (RR, 0.85; 95% CI, 0.82–0.87), and patients of Other race/ethnicity were 7% less likely to complete an AWV (RR, 0.93; 95% CI, 0.91–0.96). There was no significant difference in AWV completion between White and Asian patients.

After covariate adjustment, the probability of AWV completion was only 4% lower in Black (RR, 0.96; 95% CI, 0.93–1.00) and 5% lower in Latino/Hispanic (RR, 0.95; 95% CI, 0.91–0.99) patients than in Whites (Table 2). In contrast, Asian patients had a 4% higher adjusted probability of AWV completion than Whites (RR, 1.04; 95% CI, 1.01–1.06). There was no significant difference in adjusted AWV completion between Whites and patients of Other race/ethnicity.

**Table 2:** Adjusted Relative Risks of AWV Completion During Year 2, By Patient Characteristics

Characteristic (*N* = 92,634)	Adjusted relative risk (95% CI)
Race/ethnicity	
White non-Hispanic	(ref)
Black non-Hispanic	**0.96 (0.93–1.00)**
Latino/Hispanic	**0.95 (0.91–0.99)**
Asian non-Hispanic	**1.04 (1.01–1.06)**
Other non-Hispanic	0.99 (0.97–1.01)
Age	
65–69	(ref)
70–74	**1.03 (1.02–1.04)**^**†**^
75–79	**1.05 (1.04–1.06)**^**†**^
≥ 80	**1.02 (1.00–1.04)**
Female	1.00 (0.99–1.02)
Non-English speaker	0.95 (0.90–1.01)
Charlson comorbidity score	
0–1	(ref)
2	**1.07 (1.05–1.09)**^**†**^
3	**1.06 (1.04–1.08)**^**†**^
≥ 4	1.01 (0.99–1.03)
Dementia	**0.82 (0.79–0.85)**^**†**^
Prior year AWV completion	
No	(ref)
Yes	**1.12 (1.08–1.15)**^**†**^
Excluded	0.98 (0.94–1.03)
Medicare Advantage insurance	**0.98 (0.97–0.99)**^**†**^
Medicaid coverage	**0.93 (0.90–0.97)**^**†**^
Non-AWV primary care visits	
0	(ref)
1	**1.03 (1.01–1.05)**
2	0.98 (0.95–1.00)
≥ 3	**0.90 (0.88–0.92)**^**†**^
Patient portal used for appointment scheduling	**1.08 (1.07–1.10)**^**†**^
Health-related social needs	
0 needs	(ref)
≥ 1 need	**0.91 (0.88–0.93)**^**†**^
Not screened	**0.63 (0.61–0.65)**^**†**^

*AWV* annual wellness visit, *CI* confidence interval, *ref* referent category

* In Multivariable Poisson Regression Model With Robust Variance Estimates. Model Adjusted for All Listed Patient Characteristics, As Well As Health System Region. Generalized Estimating Equations (GEE) Used to Account for Clustering Within Primary Care Physicians; Cells in Bold Text Indicate Statistical Significance At* P* <0.05

^†^*P* < 0.01

Several covariates were positively associated with AWV completion. AWV completion during the prior year was associated with 12% greater probability of AWV completion (RR, 1.12; 95% CI, 1.08–1.15), while use of the patient portal for appointment scheduling was associated with 8% greater probability of AWV completion (RR, 1.08; 95% CI, 1.07–1.10). Compared to a low Charlson comorbidity score (0 to 1), scores of 2 (RR, 1.07; 95% CI, 1.05–1.09) and 3 (RR, 1.06; 95% CI, 1.04–1.08) were associated with higher probability of AWV completion, as was age 70 or greater (referent, 65–69).

Other patient characteristics were negatively associated with AWV completion. Compared to screening negative for social needs, screening positive was associated with 9% lower probability of AWV completion (RR, 0.91; 95% CI, 0.88–0.93), and not completing social needs screening was associated with 37% lower probability of AWV completion (RR, 0.63; 95% CI, 0.61–0.65). Dementia was associated with 18% lower probability (RR, 0.82; 95% CI, 0.79–0.85), and dual Medicaid coverage was associated with 7% lower probability (RR, 0.93; 95% CI, 0.90–0.97) of AWV completion. Compared to traditional Medicare coverage, Medicare Advantage insurance was associated with 2% lower probability of AWV screening (RR, 0.98; 95% CI, 0.97–0.99).

In each of the two sensitivity analyses, inference was identical for adjusted racial/ethnic disparities (Appendix Table 6).

## DISCUSSION

In this evaluation of a health system initiative to boost Medicare AWV completion, all racial/ethnic groups experienced similarly large increases in AWV completion, likely reflecting a highly effective initiative. However, pre-existing disparities were essentially unchanged after the 2-year initiative. Black and Latino/Hispanic patients were 15% less likely than Whites to complete AWVs in unadjusted regression analysis, but only 4% and 5% respectively less likely in adjusted analysis, while Asian patients had a higher adjusted probability of AWV completion than Whites. AWV completion was positively associated with age ≥ 70, using the patient portal for appointment scheduling, and prior year AWV completion, and negatively associated with Medicaid coverage, positive screening for any social need, and dementia.

The health system initiative led to similarly large increases in AWV uptake across all racial/ethnic groups. The initiative promoted a zero-copay service in a universally insured population, and used both in-office and out-of-office strategies to increase scheduling and completion of AWVs. Nevertheless, the lack of any meaningful reductions in disparities is noteworthy but not necessarily surprising, since this health system initiative did not include any explicit strategies to increase equity or reduce disparities.

This study has several important findings. First, it demonstrates that older adults from all racial/ethnic groups were amenable to health system efforts to boost AWV completion, leading to large increases in a preventive encounter known to offer several benefits.^10^ Second, it provides in-depth findings on racial/ethnic disparities in a population with high AWV completion, and highlights how disparities can continue to affect health services uptake, even after multiple barriers to accessing recommended care are minimized (e.g., universal insurance among Medicare populations, zero copay for AWVs). Third, it highlights correlates of racial/ethnic minority group membership that were associated with AWV completion. For example, dual Medicaid coverage and screening positive for social needs—which were more prevalent in Black and Latino/Hispanic patients than Whites—were both negatively associated with AWV completion.

Our multivariable regression analysis identified groups with low adjusted AWV completion that our team is now focusing on in a new intervention to reduce disparities, with enhanced outreach to address relevant barriers to AWV completion. Beyond Black and Latino/Hispanic patients, we are also targeting potentially high-yield subgroups including patients who did not complete AWVs during the prior year, did not use the patient portal for appointment scheduling, screened positive for social needs, or have Medicaid. After mailing letters from patients’ physicians to eligible patients, a community health worker then conducts phone outreach, where she schedules AWVs for interested patients, conducts social needs screening among those due for screening, and provides relevant resources if they screen positive for social needs.

This study has several limitations. Observational associations cannot be assumed to represent causal relationships. We partially adjusted for patient-level socioeconomic status via characteristics such as Medicaid coverage and identified social needs, but were unable to collect other socioeconomic variables that may impact AWV completion (e.g., household income, car ownership). Although some AWVs were delivered virtually following the relaxation of Medicare telehealth restrictions during the COVID-19 pandemic, pandemic-related factors may have contributed to low AWV completion rates during the baseline year (September 2020 to August 2021),^27^ and subsequent increases in AWV completion may have been partially caused by factors beyond the initiative under study. Additionally, screenings for social needs sometimes took place during AWV encounters; the observed negative association between AWV completion and not completing social needs screening was likely at least partially due to reverse causality (i.e., by not completing an AWV, patients were also less likely to have been screened). Compared to the entire state of Illinois (73.8% of older adults non-Hispanic White),^28^ the patient population under study had a lower proportion of racial/ethnic minority patients. The results observed in the health system under study may not be generalizable to other organizations and patient populations.

In conclusion, this 2-year health system initiative led to substantial increases in AWV completion in all racial/ethnic groups, but no meaningful changes in pre-existing disparities. Analyses identified multiple factors beyond race/ethnicity that were associated with AWV completion, providing insight on subgroups that could differentially benefit from future efforts, including Black or Latino/Hispanic patients, and especially those with identified social needs, dual Medicaid coverage, or nonusers of advanced patient portal functionalities.

## Supplementary Information

Below is the link to the electronic supplementary material.

Supplementary file1 (DOCX 52 KB)

## Data Availability

Datasets from the current study contain private health system data, which the authors are not currently able to share with the public research community.

## Declarations:

### Disclaimer:

The funder had no role in the design and conduct of the study; management, analysis, and interpretation of the data; preparation, review, or approval of the manuscript; or decision to submit the manuscript for publication.
