# Supplementary material for: Disparities in Medicare Annual Wellness Visits After a Systemwide Quality Improvement Initiative: A Serial Cross-sectional Analysis
Source: J Gen Intern Med. 2025 Jul 25;40(15):3755–62. doi: 10.1007/s11606-025-09773-3 (PMC12612292; doi:10.1007/s11606-025-09773-3)
Supplement: Supplementary file 1 — Supplementary file1 (DOCX 52 KB) [file 11606_2025_9773_MOESM1_ESM.docx]

Appendix Table 1: Overview of Annual Wellness Visit quality improvement intervention

| **Reasons/ underlying justification for intervention to increase AWV completion** | - AWVs known to increase preventive service completion and improve patient outcomes - AWVs are an opportunity to address quality measures that are reported to Centers for Medicare & Medicaid Services (CMS) and other payers - AWVs represent an opportunity for documentation of comorbidities and patient complexity (e.g. Hierarchical Condition Categories) that affect risk adjustment |
| --- | --- |
| **Health system teams that supported implementation of intervention** | - Clinical Operations - Clinical Documentation Improvement - Primary Care Service Line Administration - Value Based Care - Information Services/Analytics - Physician Compensation |
| **Intervention components implemented during Year 1** | - Electronic reminders sent to registered patient portal users - Opportunistic AWV scheduling at the point of care (e.g., during checkout at the end of in-person visits, clinic staff asked eligible patients if they wanted to schedule a future AWV) - Proactive identification of scheduled appointments that could be converted to an AWV |
| **Intervention components implemented during Year 2 (in addition to those from Year 1)** | - Primary care physicians received compensation bonus of up to $10,000, depending on system-level increases in AWV completion and percent of their attributed patients who completed AWVs - Continued refinement of AWV tools in Epic electronic health record to facilitate AWV delivery/documentation, e.g. created a new AWV ExpressLane tool that collapsed all required documentation in a single place to facilitate efficient completion of all required visit elements - Created reports of patients scheduled for a non-AWV visit who would be eligible for an AWV at the time of their scheduled visit, allowing for potential conversion of the scheduled visit to an AWV (if appropriate) - Created reports of patients who were due for an AWV and did not have any scheduled visits. Clinic staff conducted outreach via electronic messages and phone calls to patients listed on these reports - Developed a performance dashboard so clinical/administrative leaders could monitor progress on AWV completion. Physicians given self-service access to reports including their attributed patients |

*Abbreviations: AWV, Annual Wellness Visit*

Appendix Table 2: AWV completion during each study year, by patient characteristics

| **Characteristic** | **Pre-Intervention Year 0 (N=69,008), No. (column %)*** | | **Intervention Year 1 (N=76,652), No. (column %)*** | | **Intervention Year 2 (N=92,634), No. (column %)*** | |
| --- | --- | --- | --- | --- | --- | --- |
|  | **AWV Not Completed** | **AWV Completed** | **AWV Not Completed** | **AWV Completed** | **AWV Not Completed** | **AWV Completed** |
| *N (row %)*******^,†^* | *36,233 (52.5%)* | *32,775 (47.5%)* | *33,398 (43.6%)* | *43,254 (56.4%)* | *28,785 (31.1%)* | *63,849 (68.9%)* |
| Race/ethnicity |  |  |  |  |  |  |
| White non-Hispanic | 29,548 (81.6) | 28,120 (85.8) | 26,772 (80.2) | 36,834 (85.2) | 22,690 (78.8) | 53,679 (84.1) |
| Black non-Hispanic | 2,945 (8.1) | 1,832 (5.6) | 2,874 (8.6) | 2,504 (5.8) | 2,527 (8.8) | 3,794 (5.9) |
| Latino/Hispanic | 1,468 (4.0) | 859 (2.6) | 1,473 (4.4) | 1,233 (2.9) | 1,385 (4.8) | 2,030 (3.2) |
| Asian non-Hispanic | 919 (2.5) | 821 (2.5) | 877 (2.6) | 1,099 (2.5) | 835 (2.9) | 1,779 (2.8) |
| Other non-Hispanic | 1,353 (3.7) | 1,143 (3.5) | 1,402 (4.2) | 1,584 (3.7) | 1,348 (4.7) | 2,567 (4.0) |
| Age |  |  |  |  |  |  |
| 65-69 | 14,551 (40.2) | 10,320 (31.5) | 11,071 (33.2) | 12,792 (29.6) | 7,484 (26.0) | 16,537 (25.9) |
| 70-74 | 8,819 (24.3) | 10,192 (31.1) | 8,482 (25.4) | 13,157 (30.4) | 7,558 (26.3) | 18,839 (29.5) |
| 75-79 | 6,104 (16.9) | 6,689 (20.4) | 6,372 (19.1) | 9,130 (21.1) | 5,987 (20.8) | 14,199 (22.2) |
| ≥80 | 6,759 (18.7) | 5,574 (17.0) | 7,473 (22.4) | 8,175 (18.9) | 7,756 (26.9) | 14,274 (22.4) |
| Sex |  |  |  |  |  |  |
| Female | 21,355 (58.9) | 19,923 (60.8) | 20,040 (60.0) | 25,974 (60.0) | 17,373 (60.4) | 38,162 (59.8) |
| Male | 14,878 (41.1) | 12,852 (39.2) | 13,358 (40.0) | 17,280 (40.0) | 11,412 (39.6) | 25,687 (40.2) |
| Primary language |  |  |  |  |  |  |
| English | 35,052 (96.7) | 32,103 (98.0) | 32,147 (96.2) | 42,307 (97.8) | 27,546 (95.7) | 62,172 (97.4) |
| Non-English | 1,181 (3.3) | 672 (2.0) | 1,251 (3.8) | 947 (2.2) | 1,239 (4.3) | 1,677 (2.6) |
| Primary insurance |  |  |  |  |  |  |
| Traditional Medicare | 24,689 (68.1) | 22,206 (67.8) | 22,305 (66.8) | 29,403 (68.0) | 18,491 (64.2) | 43,390 (68.0) |
| Medicare Advantage | 11,544 (31.9) | 10,569 (32.2) | 11,093 (33.2) | 13,851 (32.0) | 10,294 (35.8) | 20,459 (32.0) |
| Medicaid coverage |  |  |  |  |  |  |
| No | 34,582 (95.4) | 32,038 (97.8) | 31,593 (94.6) | 42,178 (97.5) | 27,205 (94.5) | 61,793 (96.8) |
| Yes | 1,651 (4.6) | 737 (2.2) | 1,805 (5.4) | 1,076 (2.5) | 1,580 (5.5) | 2,056 (3.2) |
| Health-related social needs |  |  |  |  |  |  |
| 0 needs | 63 (0.2) | 31 (0.1) | 4,397 (13.2) | 7,963 (18.4) | 7,181 (25.0) | 38,730 (60.7) |
| ≥1 need | 831 (2.3) | 658 (2.0) | 825 (2.5) | 793 (1.8) | 852 (3.0) | 2,184 (3.4) |
| Not screened | 35,339 (97.5) | 32,086 (97.9) | 28,176 (84.4) | 34,498 (79.8) | 20,752 (72.1) | 22,935 (35.9) |
| Charlson comorbidity score |  |  |  |  |  |  |
| 0-1 | 9,480 (26.2) | 7,941 (24.2) | 7,305 (21.9) | 9,231 (21.3) | 5,726 (19.9) | 11,347 (17.8) |
| 2 | 11,869 (32.8) | 12,823 (39.1) | 10,585 (31.7) | 16,186 (37.4) | 8,540 (29.7) | 22,342 (35.0) |
| 3 | 6,454 (17.8) | 5,628 (17.2) | 6,295 (18.9) | 8,119 (18.8) | 5,471 (19.0) | 12,680 (19.9) |
| ≥4 | 8,430 (23.3) | 6,383 (19.5) | 9,213 (27.6) | 9,718 (22.5) | 9,048 (31.4) | 17,480 (27.4) |
| Dementia |  |  |  |  |  |  |
| No | 35,495 (98.0) | 32,357 (98.7) | 32,387 (97.0) | 42,540 (98.4) | 27,404 (95.2) | 62,319 (97.6) |
| Yes | 738 (2.0) | 418 (1.3) | 1,011 (3.0) | 714 (1.6) | 1,381 (4.8) | 1,530 (2.4) |
| Patient portal used for appointment scheduling |  |  |  |  |  |  |
| No | 22,383 (61.8) | 17,758 (54.2) | 23,654 (70.8) | 28,062 (64.9) | 20,628 (71.7) | 38,750 (60.7) |
| Yes | 13,850 (38.2) | 15,017 (45.8) | 9,744 (29.2) | 15,192 (35.1) | 8,157 (28.3) | 25,099 (39.3) |
| Non-AWV primary care visits |  |  |  |  |  |  |
| 0 | 6,882 (19.0) | 11,797 (36.0) | 6,321 (18.9) | 13,883 (32.1) | 8,495 (29.5) | 19,045 (29.8) |
| 1 | 9,394 (25.9) | 9,607 (29.3) | 8,050 (24.1) | 13,031 (30.1) | 6,993 (24.3) | 18,835 (29.5) |
| 2 | 7,899 (21.8) | 5,512 (16.8) | 7,260 (21.7) | 7,485 (17.3) | 5,258 (18.3) | 11,706 (18.3) |
| ≥3 | 12,058 (33.3) | 5,859 (17.9) | 11,767 (35.2) | 8,855 (20.5) | 8,039 (27.9) | 14,263 (22.3) |

*Abbreviations: AWV, Annual Wellness Visit*

* Percents may not sum to exactly 100% due to rounding

† Row percents presented in the row listing total numbers of patients who completed, and did not complete, AWVs. All other percents in the table represent column percents within individual patient characteristics

Appendix Table 3: Patient characteristics in first two years of serial cross-sectional analysis

| **Characteristic** | **Year, No. (column %)*** | |
| --- | --- | --- |
|  | **Pre-Intervention**  **Year 0** | **Intervention**  **Year 1** |
|  |  |  |
| *Total, N* | *69,008* | *76,652* |
| Race/ethnicity |  |  |
| White non-Hispanic | 57,668 (83.6) | 63,606 (83.0) |
| Black non-Hispanic | 4,777 (6.9) | 5,378 (7.0) |
| Latino/Hispanic | 2,327 (3.4) | 2,706 (3.5) |
| Asian non-Hispanic | 1,740 (2.5) | 1,976 (2.6) |
| Other non-Hispanic | 2,496 (3.6) | 2,986 (3.9) |
| Age |  |  |
| 65-69 | 24,871 (36.0) | 23,863 (31.1) |
| 70-74 | 19,011 (27.6) | 21,639 (28.2) |
| 75-79 | 12,793 (18.5) | 15,502 (20.2) |
| ≥80 | 12,333 (17.9) | 15,648 (20.4) |
| Sex |  |  |
| Female | 41,278 (59.8) | 46,014 (60.0) |
| Male | 27,730 (40.2) | 30,638 (40.0) |
| Primary language |  |  |
| English | 67,155 (97.3) | 74,454 (97.1) |
| Non-English | 1,853 (2.7) | 2,198 (2.9) |
| Charlson comorbidity score |  |  |
| 0-1 | 17,421 (25.2) | 16,536 (21.6) |
| 2 | 24,692 (35.8) | 26,771 (34.9) |
| 3 | 12,082 (17.5) | 14,414 (18.8) |
| ≥4 | 14,813 (21.5) | 18,931 (24.7) |
| Dementia |  |  |
| No | 67,852 (98.3) | 74,927 (97.8) |
| Yes | 1,156 (1.7) | 1,725 (2.2) |
| Primary insurance |  |  |
| Traditional Medicare | 46,895 (68.0) | 51,708 (67.5) |
| Medicare Advantage | 22,113 (32.0) | 24,944 (32.5) |
| Medicaid coverage |  |  |
| No | 66,620 (96.5) | 73,771 (96.2) |
| Yes | 2,388 (3.5) | 2,881 (3.8) |
| Non-AWV primary care visits |  |  |
| 0 | 18,679 (27.1) | 20,204 (26.4) |
| 1 | 19,001 (27.5) | 21,081 (27.5) |
| 2 | 13,411 (19.4) | 14,745 (19.2) |
| ≥3 | 17,917 (26.0) | 20,622 (26.9) |
| Patient portal used for appointment scheduling |  |  |
| No | 40,141 (58.2) | 51,716 (67.5) |
| Yes | 28,867 (41.8) | 24,936 (32.5) |
| Health-related social needs |  |  |
| 0 needs | 94 (0.1) | 12,360 (16.1) |
| ≥1 need | 1,489 (2.2) | 1,618 (2.1) |
| Not screened | 67,425 (97.7) | 62,674 (81.8) |

*Abbreviations: AWV, Annual Wellness Visit*

* Percents may not sum to exactly 100% due to rounding

Appendix Table 4: Adjusted relative risks of AWV completion during Year 2, by patient characteristics, in sensitivity analyses

| **Characteristic (N=92,634)** | **Adjusted Relative Risk (95% CI)** | |
| --- | --- | --- |
|  | **Sensitivity Analysis 1: Model Not Adjusting for Prior Year AWV*** | **Sensitivity Analysis 2: Expanded Primary Outcome Definition^†^** |
| Race/ethnicity |  |  |
| White non-Hispanic | (ref) | (ref) |
| Black non-Hispanic | **0.96 (0.93-0.99)** | **0.96 (0.93-0.99)** |
| Latino/Hispanic | **0.94 (0.91-0.98)^‡^** | **0.96 (0.92-1.00)** |
| Asian non-Hispanic | **1.03 (1.00-1.06)** | **1.04 (1.01-1.07)^‡^** |
| Other non-Hispanic | 0.98 (0.96-1.00) | 0.99 (0.97-1.01) |
| Age |  |  |
| 65-69 | (ref) | (ref) |
| 70-74 | **1.04 (1.03-1.05)^‡^** | **1.01 (1.00-1.02)** |
| 75-79 | **1.06 (1.05-1.08)^‡^** | **1.03 (1.01-1.04)^‡^** |
| ≥80 | **1.03 (1.01-1.05)^‡^** | 0.99 (0.97-1.01) |
| Female | 1.00 (0.99-1.02) | 1.00 (0.98-1.01) |
| Non-English speaker | 0.94 (0.89-1.00) | 0.95 (0.89-1.00) |
| Charlson comorbidity score |  |  |
| 0-1 | (ref) | (ref) |
| 2 | **1.08 (1.05-1.10)^‡^** | **1.07 (1.05-1.09)^‡^** |
| 3 | **1.06 (1.04-1.08)^‡^** | **1.06 (1.04-1.08)^‡^** |
| ≥4 | 1.01 (0.98-1.03) | 1.00 (0.98-1.03) |
| Dementia | **0.81 (0.78-0.84)^‡^** | **0.81 (0.79-0.84)^‡^** |
| Prior year AWV completion |  |  |
| No | -- | (ref) |
| Yes | -- | **1.08 (1.05-1.11)^‡^** |
| Excluded | -- | 0.97 (0.92-1.01) |
| Medicare Advantage insurance | **0.98 (0.97-0.99)^‡^** | **0.97 (0.96-0.98)^‡^** |
| Medicaid coverage | **0.92 (0.89-0.96)^‡^** | **0.92 (0.89-0.96)^‡^** |
| Non-AWV primary care visits |  |  |
| 0 | (ref) | (ref) |
| 1 | **1.03 (1.01-1.05)** | **1.03 (1.01-1.05)** |
| 2 | **0.97 (0.95-1.00)** | 0.98 (0.95-1.00) |
| ≥3 | **0.89 (0.87-0.92)^‡^** | **0.90 (0.87-0.92)^‡^** |
| Patient portal used for appointment scheduling | **1.09 (1.08-1.10)^‡^** | **1.08 (1.07-1.10)^‡^** |
| Health-related social needs |  |  |
| 0 needs | (ref) | (ref) |
| ≥1 need | **0.90 (0.88-0.92)^‡^** | **0.91 (0.88-0.93)^‡^** |
| Not screened | **0.63 (0.61-0.65)^‡^** | **0.63 (0.61-0.65)^‡^** |

*Abbreviations: AWV, Annual Wellness Visit; CI, confidence interval; CPT, Current Procedural Terminology; ref, referent category*

Cells in bold text indicate statistical significance at P<0.05.

* In this sensitivity analysis, we estimated the same multivariable regression model as in the main analysis, but did not adjust for Year 1 AWV completion

† In main analysis, the primary outcome for AWV completion defined as the presence of any of 3 CPT codes for Medicare preventive visits (G0402, G0438, G0439); 68.9% of patients met this primary outcome definition in Year 2. In this sensitivity analysis, outcome definition expanded to also include 2 CPT codes for non-Medicare preventive visits in older adults (99387, 99397); 70.1% met this alternative outcome definition in Year 2

‡ P<0.01
